# Colliding Wars: A Systematic Review on HIV Responses in Conflict‐Affected Settings

**DOI:** 10.1002/puh2.70236

**Published:** 2026-04-28

**Authors:** Mona Ibrahim, Alhadi Khogali, Eslam Sadeldin, Ethar Abosam, Yejide Okungbaye, Esther Osime, Mohammed F. Adam, Mohamed Elsheikh, Bothaina Eltigani, Janina Jochim, Maysoon Dahab, Rachel Yates, Lucie Cluver

**Affiliations:** ^1^ University of Oxford Oxford UK; ^2^ National Ribat University Khartoum Sudan; ^3^ Faculty of Pharmacy University of Gezira Wad Madani Sudan; ^4^ Ahfad University for Women Khartoum Sudan; ^5^ University of Lagos Lagos Nigeria; ^6^ KIT Royal Tropical Institute Amsterdam the Netherlands; ^7^ University of Science and Technology Khartoum Sudan; ^8^ St George's University School of Medicine, True Blue St George's Grenada; ^9^ Brighton and Sussex Medical School Brighton UK; ^10^ London School of Hygiene and Tropical Medicine London UK; ^11^ University of Cape Town Cape Town South Africa

## Abstract

**Background:**

Armed conflicts are an escalating threat to public health, often marked by violence, poverty, displacement and weakened health systems—conditions that mirror the drivers of HIV transmission.

**Aim:**

This review examines how armed conflict is associated with HIV vulnerability, disruptions to HIV services and how service delivery has adapted in these settings.

**Methods:**

A systematic search of six databases (MEDLINE, Embase, Scopus, CENTRAL, OVID, CINAHL) was conducted up to June 2022. Six reviewers independently screened studies, resolving discrepancies through consensus.

**Results:**

Of 7378 records, 17 met inclusion criteria. Studies revealed heightened HIV risk among adolescent girls, young women and displaced populations. Service interruptions—due to looting, supply chain breakdowns and population movement—led to treatment gaps and increased loss‐to‐follow‐up. Adolescent girls, refugees, and those living in temporary shelters experienced consistently worse HIV risks and outcomes. Despite broad search terms, there was little‐to‐no evidence on some key populations, including prisoners, sex workers and people who inject drugs. Adaptive HIV prevention and response strategies—including hybrid delivery models, integrated medical supply chains and runaway bags (emergency stock packs)—were reported as promising but under‐documented approaches.

**Conclusions:**

Conflict‐driven displacement and health system disruption are associated with heightened HIV vulnerability in some settings though effects vary by context. People living with HIV in conflict‐affected areas face disproportionate risks and must be prioritized within humanitarian response plans and in host‐country health systems.

## Graphical abstract

Armed conflicts heighten HIV vulnerability among adolescent girls, refugees and displaced populations through service disruptions and health system collapse. This systematic review of 17 studies identifies promising adaptive strategies like mobile clinics and emergency stock packs to sustain HIV care. (See the generated image.)

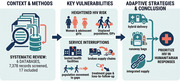

## 1 Introduction

In 2019, UNAIDS estimated that around 2.57 million people living with HIV (PLHIV) are affected by humanitarian disasters globally, most living in highly armed and conflict‐affected areas [1]. However, the impact of armed conflict on HIV incidence and prevalence is not yet fully understood. Initial theories in earlier years of this millennium suggested that countries with violent conflicts had lower levels of HIV infection in contrast with more stable counterparts, considering populations are likely ‘locked’ in one area [2, 3, 4]. More recent evidence suggests that HIV risks may be heightened in some conflict‐affected settings, although findings may vary across contexts and conflict types. A probable reason for these conflicting narratives is that armed conflicts are often associated with weakened or damaged health information systems [3]; in states with large‐scale armed violence, national trackers of HIV incidence are often suspended. However, the absence of reliable data on HIV incidence does not equal evidence of absence; available data may underestimate or mischaracterize HIV burden in conflict‐affected areas. It is also difficult to estimate HIV incidence via mathematical models, which require more stable conditions and data trends [5].

**FIGURE 1:**
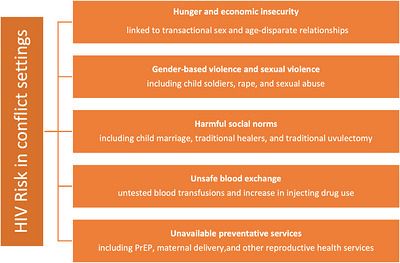
Conflict HIV: evidence‐based risk pathways.

New HIV infections can occur through unprotected sexual exposure, vertical transmission or sharing of contaminated needles, syringes or blood products—armed conflict has been noted to either directly or indirectly increase all three. This can be mediated through several risk pathways as illustrated in Figure 1 [6, 7, 8, 9, 10, 11, 12, 13, 14]. Considering the significant absence of epidemiological and surveillance data in conflict‐affected countries, the diagram in Figure 1 considers the HIV‐risk pathways on the basis of existing global literature. Although those risk pathways have been well described, they rarely recognize the implications for HIV responses in conflict‐affected settings or any possibilities for their integration into humanitarian assistance.

Armed conflict may influence HIV vulnerability through multiple interacting mechanisms, which weakens societal and psychosocial systems that safeguard communities against HIV [15] and reduces health system capacities to deliver preventative care [5]. Conversely, conflict may also suppress certain HIV‐risk behaviours in specific contexts, for example, through reduced mobility, constrained social networks, military discipline or temporary increases in humanitarian assistance.

Food security, schooling and sexual and reproductive health services, for example, are widely recognized as protective factors against the HIV epidemic, yet many often face forced closures and disruptions during times of conflict [16]. Large‐scale warfare also often disrupts wider government functions, education and social protection systems, leaving entire regions with less protection against HIV infection and other communicable diseases [17, 18].

**FIGURE 2:**
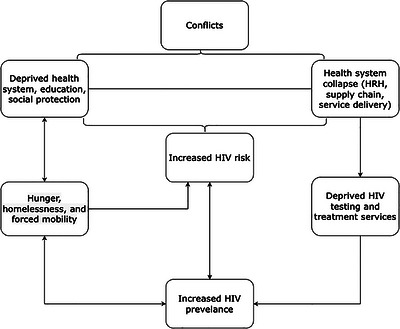
HIV risks and response in a conflict setting.

Health system disruptions in conflict‐affected settings can further compromise HIV prevention responses [19, 20]. Depending on how acute or protracted a conflict is, health systems can face serious human resource deficits, compromised supply chains and direct attacks on health facilities [21]. This could mean that PLHIV are left without testing, treatment or care services, and many conflict‐affected countries could foster an unseen HIV prevalence. PLHIV in such contexts are at heightened risks of hunger, homelessness, trauma, mental health illnesses and forced mobility—all known to promote disease progression [22] (Figure 2).

In 2010, UNAIDS called to proactively safeguard key populations (men who have sex with men, sex workers and prisoners) and women and girls in sub‐Saharan Africa, noting that these groups can be at a heightened risk of sexual violence and HIV [23, 24]. The UNAIDS Inter‐Agency Standing Committee report further emphasizes that weakened health systems in conflict‐affected countries may threaten progress already made towards achieving the three zero goals.

In 2019, the Armed Conflict Location and Event Data Project reported that globally, the largest proportion of reported sexual violence events are committed by political militias or unidentified armed groups [25]. Some vulnerable populations become further marginalized during armed conflicts, where they can face greater risks to the continuum of violence. For instance, during the war in the Democratic Republic of Congo (DRC), it was estimated that 1.8 million women and girls were victims of sexual assault [26, 27]. Additionally, HIV‐orphaned children and adolescents may also experience a disproportionate increase in HIV risk, primarily as a result of compounded economic and social adversities represented in poverty and limited access to health and education [28].

Since 2022, the number of armed conflicts in the world has increased, and the negative trend has continued in 2023 [29]. Against a background of escalating global unrest and a continuing global push towards ending the HIV epidemic, it is important to recognize the gaps in HIV response and control efforts in humanitarian contexts to ensure progress toward disease elimination. This review captures evidence on (1) the pathways through which armed conflicts may influence HIV vulnerability, including disproportionate impacts across population subgroups, (2) the disruption to HIV prevention, testing and treatment services in conflict‐affected settings, and (3) the adaptations to HIV service delivery in conflict‐affected areas. The result will inform a better understanding of HIV response needs in conflict‐affected regions, highlighting opportunities for conflict‐sensitive programming.

## 2 Methodology

### 2.1 Aim and Context

To systematically review evidence on disruptions to HIV prevention, testing, treatment and care services in conflict‐affected settings (as defined by the World Bank Group), and the documented adaptations to HIV service delivery. A systematic online search was conducted on the following databases: OVID, MEDLINE, Embase, Scopus, CENTRAL and CINAHL (search strategy in Table 1). The final search was conducted in June 2022, reflecting the timing of protocol registration and data extraction, and a 10‐year restriction was applied to the date of publication. Only records written in English were included, given the language proficiency of the authors. A protocol for this systematic review was published in the PROSPERO register, CRD42022330100 [30].

**TABLE 1:** Search strategy.

	AND	AND	AND
Afghanistan Somalia Syria Yemen Armenia Azerbaijan Burkina Faso Burundi Cameroon Central African Republic Chad Congo Iraq Libya Mali Mozambique Myanmar Niger Nigeria Eritrea Guinea‐Bissau Kosovo Lebanon Papua New Guinea Sudan Venezuela, RB Gaza Zimbabwe South Sudan	International armed conflict Non‐International armed conflict War Armed conflict* Conflict* War Violence Violent conflict* Post conflict Rape Humanitarian emergency* Emergency* Humanitarian Internally Displaced Refugees Insecurity	‘People affected by HIV’ ‘at risk of HIV’ PLHIV ALHIV AIDS‐affected populations ‘HIV‐affected’ ‘HIV affected’ ‘HIV infected’ ‘ART adherence’ ‘Vertical infection’ ‘Antiretroviral adherence’ ‘child* HIV’ PMTCT ‘Men who have sex with men’ MSM Transgender Prison inmate* Prisoner* Detainee* ‘Sex workers’ ‘Adolescent girls’ ‘IV drug user’	‘HIV incidence’ ‘Sexual violence’ ‘High‐risk behaviour’ ‘High‐risk behaviour’ ‘High‐risk sex’ ‘HIV testing’ ‘Access to treatment’ ‘Access to ART’ SRHR SRH ‘Sexual health’ ‘Reproductive health’ ‘Service interruption’ ‘Loss to follow‐up’ ‘Loss to care’ ‘ART non‐adherence’ ‘HIV risk’ ‘Condom distribution’ ‘Child* HIV’ ‘Transactional relationships’ ‘Transactional sex’ ‘Drug abuse’ ‘Substance abuse’ ‘Who inject drugs’

### 2.2 Design and Inclusion Criteria

All studies analysing HIV responses in conflict‐affected countries were included, detailing any difference in HIV risk and changes in HIV testing, antiretroviral adherence, and viral suppression in these settings. For a thorough review, no methodological restriction was applied. Studies with incomplete data and those from high income countries, or countries without an ongoing conflict were excluded. Last search was done on 3 June 2022 (Table 2).

**TABLE 2:** Database searches and results.

Database	Number of articles retrieved
OVID	1745
MEDLINE	5134
Embase	111
Scopus	910
CENTRAL	138
CINAHL	318

**FIGURE 3:**
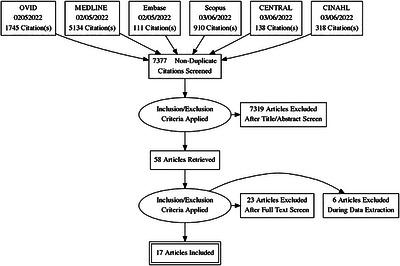
PRISMA flowchart of the study selection process.

Using the Rayyan platform (https://rayyan.ai), six independent reviewers (divided into three paired groups) screened abstracts of retrieved records for potential inclusion then independently reviewed record full‐texts for final inclusion. Conflicting decisions between them were resolved by discussion and consensus. Study selection was guided by the Preferred Reporting Items for Systematic Reviews and Meta‐Analyses (PRISMA) guidelines [31], as demonstrated in Figure 3.

A data extraction sheet was used to collect relevant findings from the included studies—including study type, country, time, duration of conflict and key outcomes, such as: reported increases in immediate causes of HIV; reported changes in testing and treatment services; reports on delayed testing, antiretroviral therapy (ART) non‐adherence and loss to follow‐up (LTFU); disproportionate impacts of conflict on sub‐populations. Risk of bias assessment was done to studies, and records with high risk of bias were excluded. In total, 17 articles were included in this review (Table 3).

**TABLE 3:** Characteristics of the included studies.

Study code	Country (s)	Method	Primary objective	Type of conflict	Outcomes measured	Main findings
Abdullahi et al. [32]	Nigeria	Cross‐sectional survey	To determine the impact of TB case‐finding interventions on notifications in an intervention area compared with historical and contemporary controls	Armed conflict	Reported increases in immediate causes of HIV (high‐risk sex and IV drug use); disproportionate impacts of conflict on key populations, children born to HIV mothers, and adolescent girls	Disproportionately higher HIV incidence in women; roadblocks are mitigated via multidisciplinary collaboration and coordination; testing campaigns substituting failing lab has been useful in detecting cases
Bermudez et al. [33]	Ethiopia	Cross‐sectional study	Examine the associations between HIV risk factors, attitudes on gender inequality, IPV acceptability, and self‐esteem for female adolescent refugees	Armed tribal conflict	Reported increases in immediate causes of HIV (high‐risk sex and IV drug use)	Increased transactional sex, child marriage, intimate partner violence and unprotected sex in conflict settings
Bress et al. [34]	DRC	Quasi‐experimental	Evaluate a post‐rape medical care in remote and resource‐limited settings through stock monitoring	Armed state conflict	Reported increases in immediate causes of HIV (high‐risk sex and IV drug use), Reported changes in testing and treatment services	Increased GBV during conflicts; rape and emergency HIV exposure testing and therapy functioned despite conflict
Buju et al. [40]	DRC	Cohort study	To identify predictors of viral failure (or viral non‐suppression) among HIV‐infected patients under a DTG‐based regimen in the context of ongoing armed conflict since	Armed state conflict	Reported increases in immediate causes of HIV; Reports on delayed testing, ART non‐adherence, and loss to follow‐up	Ethnicity and age were found to correlate with viral non‐suppression and non‐adherence; young girls are used as sexual objects
Buju et al. [47]	DRC	Cohort study	Examine the incidence and predictors of LTFU in the context of ongoing atrocities caused by armed conflict	Armed state conflict	Reports on delayed testing, ART non‐adherence, and loss to follow‐up	Conflict was associated with increased LTFU
Crellen et al. [36]	Central African Republic	Prospective cohort study	To estimate the impact of the conflict on HIV patient's mortality	Armed state conflict	Reported increases in immediate causes of HIV (high‐risk sex and IV drug use), reported changes in testing and treatment services	Males had more odds of HIV mortality in conflict settings. Conflicts were associated with damage in laboratory equipment
Ferreyra et al. [46]	South Sudan	Semi‐qualitative cohort	To determine the feasibility and outcomes of community‐based HIV interventions in a conflict setting	Armed tribal conflict	Reports on delayed testing, ART non‐adherence, and loss to follow‐up	Conflict did not necessarily affect HIV testing capacity in conflict areas, but referral services were not intact Digital technologies helped in sharing information about ARV stocks
Ferreyra et al. [42]	Yemen, CAR	Case‐cohort	To summarize the experience and results of providing ART and implementing contingency plans during acute instability	Violence (CAR), War (Yemen)	Reported changes in testing and treatment services, Reports on delayed testing, ART non‐adherence, and loss to follow‐up	LTFU rates due to conflicts varied across countries; HIV responses in conflict‐affected settings can be sustained with simplified models and contingency plans; vertical and internationally‐funded HIV intervention is less affected by conflict
Haddison et al. [39]	Cameroon	A retrospective survey	To assess the utilisation of health services before and during the armed conflicts	Armed conflict	Reported changes in testing and treatment services; loss to follow‐up	Health facilities lost more than half of their workforce; LTFU doubled during acute conflict
Kaboru et al. [45]	DRC	Cross sectional	Assessing the provision of HIV/TB co‐infection services in health facilities in the conflict	Humanitarian crisis	Reported increases in immediate causes of HIV (high‐risk sex and IV drug use); reported changes in testing and treatment services, reports on delayed testing, ART non‐adherence, and loss to follow‐up	Conflict led to service disruption and fragmentation; High HIV prevalence amongst soldiers; reported loss of referral systems to HIV services, e.g., Tuberculosis and Hepatitis C clinics
Mayada Faisal Nabih et al. [44]	Yemen	Retrospective descriptive study	To examine demographic data of people living with human immunodeficiency virus (PLHIV) who are LTFU during HIV treatment and care	Intermittent armed conflicts	Reported changes in testing and treatment services, Reports on delayed testing, ART non‐adherence, and loss to follow‐up	Conflict led to HIV services disruption in numerous districts; impact of conflict on HIV service also affected nearby ‐relatively stable‐ areas
Mude and Nyanhanda [43]	South Sudan, CAR, DRC, Sudan, Ghana, Nigeria, Zimbabwe	Cross‐sectional analysis	To assess HIV testing rates during antenatal care (ANC) in seven sub‐Saharan African countries	Various types of conflict	Reported changes in testing and treatment services comparing five countries with different levels of armed conflict and fragility	HIV testing and treatment is notably lower in countries experiencing higher levels of armed conflict
Omam et al. [38]	Cameroon	Quasi‐experimental cohort	To evaluate the mobile clinics model to identify best practices in piloting the implementation of integrated HIV DSD in fragile and conflict‐affected settings	Armed civil conflict	Reported changes in testing and treatment services, Reports on delayed testing, ART non‐adherence, and loss to follow‐up	Health seeking behaviour is affected by increased distance between displacement camps and HIV care centres; lockdowns and arm confrontations rendered several HIV services inaccessible due to security constraints; using mobile clinics might be more effective, especially when linked to a nearby facility thus reducing LTFU
Rieger [35]	Burundi	Cross‐sectional analysis	To study the relationship between civil war and HIV/AIDS in Burundi at the micro level	Civil war	Reported increases in immediate causes of HIV (high‐risk sex and IV drug use)	Increased GBV during conflicts; HIV and STIs are higher amongst HIV patients
Ssonko et al. [41]	CAR, DRC, South Sudan	A descriptive analysis	Review the implementation of differentiated HIV care and treatment approaches in MSF‐supported programmes in three African countries	Various types of conflict	Reported changes in testing and treatment services, reports on delayed testing, ART non‐adherence, and loss to follow‐up	Attacks to health care facilities leads to disrupted service provision temporarily; LTFU cases increase after a health facility is attacked
Todd et al. [37]	Afghanistan	Cohort study	To measure incidence and potential predictors, including environmental events and needle and syringe distribution and collection program (NSP) use, of hepatitis C virus (HCV) and HIV among IDUs	Insurgent attacks	Reported increases in immediate causes of HIV (high‐risk sex and IV drug use)	Increased IV drug use during conflicts; conflict leads to facility closure, reduction in counselling services and increased HIV and HCV risks
Yoder et al. [56]	Kenya	Retrospective analysis	describe both the immediate and long‐term impact of this conflict for HIV‐infected children	Post‐election conflict	Reports on delayed testing, ART non‐adherence, and loss to follow‐up; Disproportionate impacts of conflict on key populations, children born to HIV mothers, and adolescent girls	Armed conflicts leads to increased service disruptions

Abbreviations: DRC, Democratic Republic of Congo; LTFU, loss to follow‐up.

## 3 Results

### 3.1 Pathways Influencing HIV Vulnerability in Conflict‐Settings

The eight articles addressing this theme emphasized increased unprotected sexual exposure as a key feature in conflict settings. The articles detailed HIV‐risk pathways in the following section.

#### 3.1.1 Pathways of Increased Sexual Transmission via Sexual Violence and Harmful Gender Norms

HIV risk was reported to be disproportionately higher in women affected by conflict. In a Nigerian conflict zone, for example, females comprised 46.3% of those tested for HIV yet accounted for 60.6% of HIV‐positive results; whereas the average HIV prevalence in the community was 1.5%, which was around 1.9% in females and 1.1% in males [32]. Another study conducted in an Ethiopian refugee camp and temporary settlements validated this increased HIV risk among displaced girls and young women. Specifically, those who adopted inequitable gender norms and expressed high‐IPV acceptability were found to be at greater risk of ever experiencing forced (OR 1.40, CI 1.15–1.70; OR 1.66, CI 1.42–1.94) or transactional sex (OR 1.28, CI 1.05–1.55; OR 1.59, CI 1.37–1.85) [33]. Findings from DRC and Burundi complement this risk pathway, highlighting rampantly increased gender‐based violence and sexual violence (including intimate partner violence) during conflict [34, 35]. However, another research article in Central African Republic demonstrates that males living with HIV have 70% higher odds of mortality compared to their female peers—indicating possible gendered difference between HIV mortality and risk of infection [36]. It is clear that there are differentiated HIV‐risk pathways across genders; however, the extent and severity of these risks differ depending on the context.

#### 3.1.2 Evidence on HIV Intravenous (IV) and Vertical Transmission

Injecting drug use is commonly known to increase the risk for HIV. Our search identified very limited empirical evidence on people who inject drugs in conflict‐affected settings, likely reflecting barriers related to criminalization, stigma, insecurity and disrupted surveillance systems; however, one study tested a programme for needle and syringe distribution in Afghanistan, which demonstrated that there are overlapping risks between HIV and Hepatitis C infections among needle users [37]. Notably, building on longitudinal data, the study emphasizes that armed conflict exacerbations cannot independently predict infection risk. Our search did not capture any empirical evidence on vertical HIV transmission, transfusion statistics or comparative research comparing injecting drug use in conflict zones to more stable regions. Similarly, although disruption to reproductive and maternal care services was often mentioned, this search did not find any results that focused on vertical HIV transmission.

#### 3.1.3 Evidence on Disproportionately Affected Groups

In Northeast Nigeria, isolated testing campaigns among refugees and in camps for Internally Displaced People (IDPs) detected a much higher prevalence of HIV and Tuberculosis compared to national prevalence rates, which were not detected through public testing facilities [32], although causality cannot be attributed solely to conflict due to the absence of comparable pre‐conflict baseline data. Our search did not yield any results about sex workers, prisoners or any other key populations.

### 3.2 Disruption to Health and HIV Services

#### 3.2.1 Getting to Health Facilities

There were several barriers to reaching HIV services in conflict‐affected regions, including the reduced independent movement of sub‐populations like adolescent girls and women; political unrest and road blockades preventing access to HIV treatment and care sites; militarised blockades and high‐security phases banning civilian movement; and increased distance between displacement camps and HIV care centres [38, 39]. In Cameroon, for example, lockdowns and arms confrontations rendered several HIV services inaccessible due to security constraints [38].

Discriminatory barriers to access are also reported among minorities in several conflict‐affected contexts. In DRC, both ethnicity and age were found to correlate with viral non‐suppression, with Sudanese‐born refugees and adolescents having high rates of LTFU and significantly faster disease progression, especially after 3 months of treatment initiation [40].

##### 3.2.1.1 Testing and Treatment Facilities

Nine studies reported changes in HIV testing or treatment services due to conflict. These interruptions were most notably due to supply chain disruptions, unavailable medicines, tests and out‐of‐stock ARTs [37, 38, 41, 42]. An analytical study comparing five countries with varying levels of conflict concluded that testing and treatment are notably lower in countries experiencing higher levels of armed conflict [43]. In Yemen, five governorates (out of 22) have provided HIV treatment and care services since 2007; although these public centres continued to function through armed exacerbations, there was a notable spike in LTFU in 2011 and 2012—coinciding with widespread political violence—this suggests that even when HIV services remain intact, there are considerable barriers to seeking care [44]. Health facilities providing HIV services were also vulnerable to damage and looting in conflict areas, detailing outcomes like damaged equipment and displacement and attrition of the health workforce [36, 39, 41]. These consequences extend to residents of both non‐conflict areas and conflict areas; in Yemen, PLHIV in relatively stable locations were also unable to access HIV services [44].

Generalized health system fragility can also have a domino effect on HIV services and campaigns. Routine antenatal HIV testing, for example, is highlighted as an essential point‐of‐care gateway for early HIV diagnosis; a recent study concluded that HIV testing in antenatal care visits becomes significantly more complex in conflict‐affected settings, with women facing barriers to reaching clinics (especially those living in rural areas) [43]. Other health services that are not specific to HIV reported a loss of referral systems to HIV services; for instance, Tuberculosis and Hepatitis C clinics that continued to function during conflict stopped testing for co‐infection with HIV [37, 45]. This was particularly relevant in DRC, for example, where 61% of Tuberculosis facilities continued to operate in conflict compared to only 9% of HIV clinics [45].

Protracted conflicts appear to have less sudden disruptions on testing and treatment systems; in DRC, rape and emergency HIV exposure testing and therapy functioned despite conflict, with a 5‐year longitudinal analysis showing timely and consistent emergency prophylaxis without medication stock‐out [34]. Evidence from CAR demonstrates that testing and treatment outcomes were similar in both conflict and stable regions; however, notably both regions had suboptimal outcomes [42]. Similarly, although DRC's health service delivery remained intact in long‐term conflict, authors also flagged that there was increased fragmentation between HIV and Tuberculosis services—deeming them an uncommon provision to begin with regardless of conflict [34].

##### 3.2.1.2 Link to Care, Support and Follow‐Up

Several public services were reported to be affected in conflict‐affected settings, including communication networks, referrals to welfare and food support, counselling and surveillance systems—notably interrupting the continuum of care [33, 41, 46]. In DRC, for example, 24% of health facilities reported some coordination with disease control programmes, only a fraction of which could manage HIV and Tuberculosis co‐infections. The study further reports that HIV testing and screening were often done in separate health facilities—dissolving one‐point‐care systems [40]. Evidence also suggests that ART follow‐up is much less likely after a health facility is attacked, despite unlikely odds of reoccurrence [41].

Ten studies indicated a spike in LTFU in conflict settings. LTFU ranged from 9% to 28.8%. For example, in DRC around 29% were LTFU during times of conflict, in Yemen, 9%, and 25% in CAR [42, 47]. When compared to the baseline in Cameroon, LTFU increased by 1.5 folds during the acute conflict in 2017 [39].

### 3.3 Adaptations to Service Delivery in Conflict‐Affected Areas

In order to circumvent these drawbacks, a study from Yemen and CAR concluded that HIV responses in conflict‐affected settings can be sustained with simplified models and contingency plans that allow emergency responsiveness [42]. MSF‐led research in South Sudan suggests that building community‐based treatment platforms and engaging community health workers can enable testing and treatment to continue when public facilities are closed during conflict exacerbations, reaching similar levels of testing and ART retention [48]. They emphasized, however, that referral systems (e.g., psychosocial support) do not necessarily remain intact [46]. Mobile clinics were also cited as alternative routes to reaching compromised settings (especially rural areas); highlighting their role in linking to nearby treatment centres to initiate and adhere to ARTs [43]. Telecommunication and innovative technologies were proven useful in HIV case tracking in some contexts, especially in countries that already had high coverage of e‐services like Yemen, although they were less successful in countries with militarized telephone systems like CAR [42]. Helplines and digital technologies can also inform PLHIV of stock availability and safe locations for follow‐up [46].

A common theme across the literature was the reported suspension of sexual and reproductive health services, vaccination services and other basic health functions (especially HIV‐preventative functions). However, evidence from MSF Yemen and CAR suggests that, despite public system failure in acute conflict, vertical and internationally funded interventions are less affected by conflict, with campaigns maintaining ART access and retention rates similar to those living in stable contexts—indicating that it is possible to maintain full functions of HIV responses with steady funding streams [42].

There is also notable evidence of LTFU prevention in conflict‐affected areas, especially through community‐level actors, two notable examples were emergency stock packs/ ‘run‐away bags’, including ART cover for 3 or 4 months for highly mobile populations, and multi‐site mobile clinics offering HIV screening and treatment services to re‐link LTFU cases to nearby HIV facilities. In Cameroon, for example, mobile clinics linked more than a third of LTFU to HIV care centres and reinitiated ART [38, 42].

## 4 Discussion

This review examined how armed conflict affects HIV service delivery and identified substantial gaps in the evidence base, particularly regarding service continuity and feasible research designs in insecure settings. It was clear that there is insufficient research comparing conflict and stable contexts, little evidence on incidence and prevalence, and the absence of randomized trials reflects the ethical and logistical constraints of conducting experimental research in conflict‐affected settings. Most of the findings in this analysis have indicated changes or adaptations made to HIV responses in conflict‐affected settings. However, there is little evidence on the accessibility of testing before conflict across these settings, so it is also possible that HIV service provision was already limited but worsened through armed exacerbation. Future research should prioritize longitudinal cohort studies, implementation research and mixed‐methods designs that are feasible in rapidly changing and insecure contexts.

The hypothesized causal pathways remain subject to interpretation; although some scholars detailed that security concerns are the primary constraint on service delivery, others have suggested that the corresponding economic shocks may exert a more profound effect on HIV responses. There is also a deficit in the available evidence on IV transmission, care for adolescents living with HIV in contexts of conflicts, and estimating HIV incidence and prevalence.

Beyond HIV services, it is also apparent that the wider state of the health system can affect both HIV‐risk mitigation and treatment continuity—with overlapping supply chains, health workforce and laboratory facilities. Consequently, preventing and responding to HIV in conflict‐settings should consider the following dimensions for wider health system strengthening in acute conflict:

1. Increase opportunities for testing and treatment: Conflict often pushes the health system to more siloed operation plans; clinics that were testing for Hepatitis C in DRC, for example, were not also testing for co‐infection with HIV. However, it is unclear whether this is a result of conflict or a feature of fragile health systems (which may occur in stable, but low‐income, countries). This was a common trend across conflict‐affected countries, likely due to the prioritization of emergency health functions [40, 49]. Similarly, literature suggests that referral systems are not usually considered a core function for health facilities. This decline in point‐of‐care systems can effectively impede HIV testing and treatment, even for PLHIV who seek care for a coexisting health condition.
2. Multi‐disciplinary partnership to preserve the medical supply chain: Roadblocks and security concerns were major impediments to the medical supply chain (including tests, PrEP and ART) and are key operational challenges in almost all the literature. However, sometimes they are disproportionately interrupted compared to other medical supply chains—for example, in some settings, Tuberculosis medications were delivered despite the blockades, and ART testing kits were not [32]. This suggests a need for infrastructure assessments to recognize and leverage supply chains that remain intact despite the conflict. For example, it is possible to safeguard HIV supplies by leveraging established food, immunization and humanitarian supply chains [48].
3. Regional follow‐up and case tracking: Forced displacement was reiterated as one of the core drivers of LTFU. Although MSF highlighted the need for medical packages for populations at risk of forced displacement, e‐interventions can further expand the network of support available for displaced PLHIV, a majority of whom are retained through digital coverage [49]. Evidence on online peer‐support groups, positive parenting support, mobile cash and care support, and e‐connections to SRH clinics should be considered in alignment to explore the possibility of combined support packages for refugees and IDPs [49, 50, 51]. Recent evidence also emphasizes the importance of monitoring pharmacy records to capture treatment interruptions and prevent LTFU [52].
4. HIV‐sensitive humanitarian responses: HIV responses can be embedded within humanitarian and emergency plans. For example, this could include (1) integrating sexual violence prevention and response across humanitarian programming (especially for key populations, those living in temporary shelters and adolescent girls) [53]; (2) increasing testing and treatment availability in mobile clinics; (3) training non‐specialist healthcare workers and community health workers to provide HIV services; and (4) increasing inclusion and outreach to refugees and IDPs—as highlighted in recent Global Fund and UNHCR joint reports [52].

Due to the massive heterogeneity in the type of conflicts, it is also apparent that high‐risk populations should be defined locally. Although there is evidence that key populations are at disproportionately higher risks of contracting HIV and not receiving appropriate care, this study has also identified that adolescent girls and young women, refugees and IDP can face similar adversity, especially in armed conflict settings [54, 55]. High‐risk groups need to be defined and adjusted according to the context.

Strengths of this review include a broad multi‐database search strategy and inclusion of diverse conflict settings, whereas limitations include language restrictions, exclusion of high‐income conflict‐affected countries and reliance on observational studies.

## 5 Conclusion

Our summary of the available evidence indicates that HIV‐risk pathways may be amplified during conflicts; however, it is unclear whether that translates to increased incidence. Multi‐pathway interruptions to HIV control are common in conflict‐affected settings, especially armed conflict. Health system interruptions, compromising access to testing and treatment, were reportedly driven by armed attacks, supply chain interruptions and specialized workforce deficits. LTFU is further complicated by displacement and forced migration. Mobile clinics and telecommunications can evidently reduce mass LTFU in conflict settings.

## Author Contributions

**Mona Ibrahim**: conceptualization, methodology, data curation, investigation, formal analysis, validation, supervision, funding acquisition, visualization, project administration, resources, writing – original draft, writing – review and editing. **Alhadi Khogali**: conceptualization, methodology, data curation, investigation, formal analysis, validation, supervision, funding acquisition, visualization, project administration, resources, writing – original draft, writing – review and editing. **Eslam Sadeldin**: conceptualization, methodology, investigation, formal analysis, writing – original draft. **Ethar Abosam**: conceptualization, methodology, investigation, formal analysis, writing – original draft. **Yejide Okungbaye**: conceptualization, methodology, investigation, formal analysis, writing – original draft. **Esther Osime**: conceptualization, methodology, investigation, formal analysis, writing – original draft. **Mohammed F. Adam**: conceptualization, methodology, investigation, formal analysis, writing – original draft. **Mohamed Elsheikh**: conceptualization, validation, supervision. **Bothaina Eltigani**: validation, writing – review and editing. **Janina Jochim**: validation, writing – review and editing. **Maysoon Dahab**: validation, supervision. **Rachel Yates**: validation, supervision, resources, writing – review and editing. **Lucie Cluver**: validation, supervision, funding acquisition, writing – review and editing.

## Ethics Statement

This study is a systematic review of previously published literature. No primary data were collected from human participants, and therefore, institutional ethics committee approval was not required. The review was conducted in accordance with PRISMA guidelines, and the protocol was prospectively registered in PROSPERO (CRD42022330100).

## Conflicts of Interest

The authors declare no conflicts of interest.

## Figures and Tables

**FIGURE A1:**
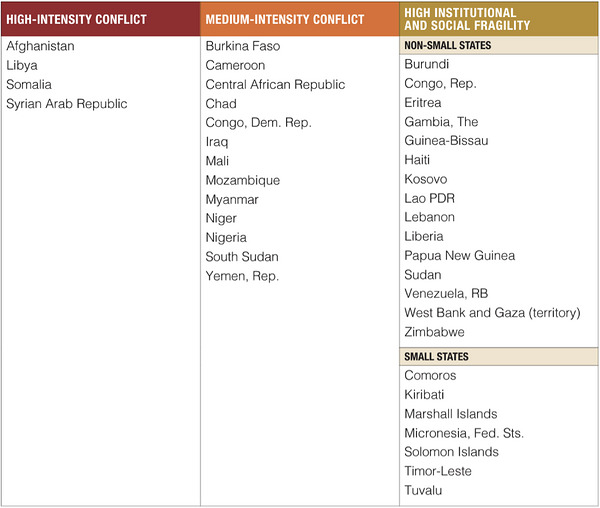
World Bank List of fragile and conflict‐affected situations.

## Data Availability

The data that support the findings of this study are available from the corresponding author upon reasonable request.

## 1

Figure A1 and Tables A1 and A2.

**TABLE A1:** Risk of bias assessment for cohort studies.

	Study and year of publication								
Cohort quality assessment using Newcastle Ottawa Scale (NOS) criteria	Bress et al. [34]	Buju et al. [40]	Buju et al. [47]	Crellen et al. [36]	Ferreyra et al. [46]	Ferreyra et al. [42]	Omam et al. [38]	Todd et al. [37]	Yoder et al. [56]
**A. Selection (maximum of four stars)**									
1. Representativeness of the exposed cohort	★	★	★	★	★	★	★	★	★
2. Selection of the non‐exposed cohort	☆	★	★	☆	★	★	☆	☆	☆
3. Ascertainment of exposure	★	★	★	★	★	★	★	★	★
4. Demonstration that outcome of interest was not present at start of study	★	★	☆	★	☆	★	★	★	★
**B. Comparability (maximum of two star)**									
1. Comparability of cohort on the basis of the design and/or analysis	☆★	★★	★★	☆★	★★	★★	☆★	☆★	☆★
**C. Outcome (maximum of three stars)**									
1. Assessment of outcome	★	★	★	★	★	★	★	★	★
2. Was follow‐up long enough for outcomes to occur?	★	★	★	★	★	★	★	★	★
3. Adequacy of follow‐up of cohorts	★	★	★	★	★	★	★	★	★
**Total (maximum of nine stars)**	7	9	8	7	8	9	7	7	7

Key: ★ (criteria fulfilled) ☆ (criteria not fulfilled).

**TABLE A2:** Risk of bias assessment for other quantitative studies.

	Studies (year)							
Cross‐sectional studies quality assessment using Newcastle Ottowa Scale (NOS) criteria	Abdullahi et al. [32]	Bermudez et al. [33]	Haddison et al. [39]	Kaboru et al. [45]	Mayada Faisal Nabih et al. [44]	Mude and Nyanhanda [43]	Rieger et al. [35]	Ssonko et al. [41]
**A. Selection (maximum of three stars)**								
1. Representativeness of the sample	★	★	★	★	★	★	★	★
2. Non‐respondents	★	☆	★	☆	★	☆	☆	★
3. Ascertainment of exposure	★	★	☆	★	★	★	★	★
**B. Comparability (maximum of two stars)**								
1. The subjects in different outcome groups are comparable, based on the study design or analysis. Confounding factors are controlled	☆☆	★★	★☆	☆☆	☆☆	★★	★★	☆★
**C. Outcome (maximum of two stars)**								
1. Assessment of outcome	★	★	★	★	★	☆	★	★
2. Statistical test	☆	★	☆	★	☆	★	★	☆
**Total (maximum of seven stars)**	4	6	4	4	4	5	6	5

Key: ★ (criteria fulfilled) ☆ (criteria not fulfilled).
